# Effect of tourist activity on wastewater quality in selected wastewater treatment plants in the Balearic Islands (Spain)

**DOI:** 10.1007/s11356-024-32173-9

**Published:** 2024-01-30

**Authors:** Joselin S. Rodríguez-Alcántara, Noelia Cruz-Pérez, Jesica Rodríguez-Martín, Alejandro García-Gil, Juan C. Santamarta

**Affiliations:** 1 Departamento de Ingeniería Agraria y del Medio Natural, Universidad de La Laguna (ULL), San Cristobal de La Laguna, Tenerife Spain; 2 Departamento de Técnicas y Proyectos en Ingeniería y Arquitectura, Universidad de La Laguna (ULL), San Cristobal de La Laguna, Tenerife Spain; 3 Geological Survey of Spain (IGME), Spanish National Research Council (CSIC), Madrid, Spain

**Keywords:** Wastewater management, Climate change, Sewage, Mediterranean climate, Seasonal tourism

## Abstract

Unregulated sewage discharge into the sea poses a considerable danger to marine ecosystems, with coastal regions being particularly vulnerable to this because of the impact of tourism. This issue is amplified during the summer season, as the Balearic Islands are a heavily frequented destination. This study aims to determine the water quality in five different wastewater treatment plants (WWTPs) representative on the islands. For this purpose, we analysed several parameters, including biochemical oxygen demand (BOD), chemical oxygen demand (COD), treated water flow, suspended solids (SS), nitrates (N) and phosphorus (P), at the inlet and outlet of the WWTPs for 5 years. We set particular thresholds for each parameter and documented any breach by comparing the findings with the existing regulations. The least favourable results indicate non-compliance regarding N and P levels throughout the entire study period, as well as a lack of reduction percentage. Furthermore, flow analysis reflects the significant influence of tourism on water quality, with notable increases in both population and treated water volume during the peak tourist season. Overall, the investigation offers a robust foundation for comprehending water quality in relation to coastal landscape in the Balearic Islands. It pinpoints significant worry spots and underscores tourism’s immediate impact on this ecological feature.

## Introduction

Island tourism offers a unique combination of environmental, landscape and cultural factors that make it attractive to visitors from all over the world. It has turned out to be a pivotal source of revenue and job creation in island communities (Chao and Chao 2017; George and Reid 2005). Nevertheless, the mounting popularity of island tourism has brought about vehement debates and environmental apprehensions. The subsequent economic growth has been a significant development in these regions (Phong and Van Tien 2021; Stevanović et al. 2022).

The rapid expansion of the industry has placed significant strain on scarce natural resources, particularly freshwater (Comerio and Strozzi 2019). The availability of drinking water and wastewater handling have emerged as crucial hurdles in numerous islands, as demand surges for water for human consumption and tourist activities; infrastructure has also grown considerably (Di Nica et al. 2021). This situation has caused overconsumption of this resource, which jeopardizes the viability of the entire ecosystem.

The impact of island tourism on the environment extends beyond the depletion of water resources. It results in degradation of marine ecosystems to allow for the development of tourist infrastructure like jetties, resorts and artificial beaches. The pollution emitted during tourism activities harms water quality, jeopardising the health of marine ecosystems and the variety of living species they support (Liamlaem et al. 2019; Wells et al. 2016).

Tourism on islands also has socio-cultural implications (Perles et al. 2023). The substantial number of tourists can modify the social and cultural dynamics of local communities and, occasionally, diminish local customs and create friction between residents and visitors. In addition, overuse of natural resources and infrastructure can cause traffic congestion and escalate living expenses for local inhabitants, which could ultimately have an adverse impact on their quality of life (Arini and Mardianta 2022; Gössling and Peeters 2015).

Climate change will also have an impact on the pressure that tourism places on water management. The effects of climate change are very diverse and will affect ecosystems, people and their way of life (Aparício et al. 2022; Lemesios et al. 2016; Milanese et al. 2011). Rising temperatures, rising sea levels, increased concentrations of greenhouse gases and an increase in extreme weather events are the main effects published in the reports of the Intergovernmental Panel on Climate Change (IPCC) (2023). Some of the consequences in the different scenarios foreseen by the IPCC will be the availability and quality of water and the increased frequency and intensity of extreme hydrological phenomena such as droughts and floods (Gössling and Peeters 2015; Köberl et al. 2015). Spain is a heterogeneous territory, and coastal cities with a Mediterranean climate or islands will be one of the most affected by this phenomenon (Lemesios et al. 2016). For this reason, the Balearic Islands are particularly vulnerable, due to their insularity, tourism-based economy and scarcity of water (Amorós et al. 2009). In this context, the integrated management of water resources becomes vital in regard to preventing and mitigating these effects (GWP 2004). Within the management of water resources, one of the fundamental phases of action is the sanitation and purification phase; energy efficiency in treatment plants can be improved, by promoting the reuse of wastewater and in innovation and technology transfer in the water sector (Sala-Garrido et al. 2011).

The wastewater treatment process aims to eliminate the pollutant load of wastewater produced in the urban, agricultural, industrial and tourism sectors by means of a series of physical, chemical and/or biological processes to then return it to the environment in the best conditions or reuse it in different areas, such as, for example, irrigation of green areas or cleaning of public areas (Gil et al. 2013; Kitajima et al. 2014). In the case of the Balearic Islands, treated water that cannot be reused is returned to aquifers or is discharged into streams, evaporation ponds and the sea. This discharge has been one of the main sources of pollution and degradation of ecosystems in the islands. If poorly treated water is discharged, it would cause serious pollution problems on the coasts of the region (Gomila et al. 2008). Similarly, in some aquifers, the rate of water extraction is higher than the rate of recharge, which has led to the intrusion of seawater into these bodies of water (Babu et al. 2018; Werner et al. 2013).

**Fig. 1:**
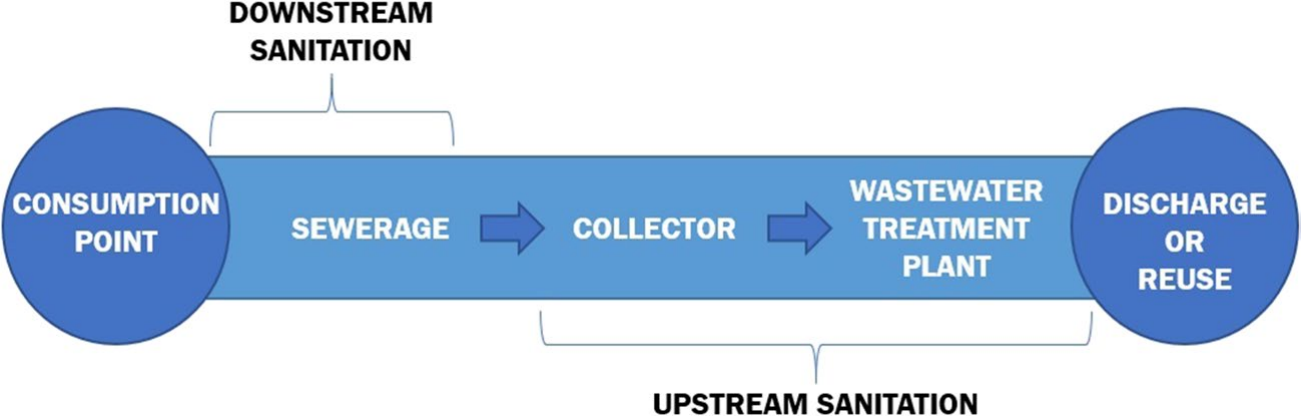
Diagram of the municipal wastewater transport and treatment process. Source: adapted from ABAQUA

In order to analyse water quality in the treatment plants, it is essential to have a thorough understanding of the sanitation and purification system operating on the islands. This competence is governed by the Balearic Water and Environmental Quality Agency (ABAQUA 2021). The system operates in two phases (Fig. 1). The initial phase, referred to as downstream sanitation, comprises collecting water from its point of use conveying it to the collector network, managed by the local sewerage system. The second phase, referred to as upstream sanitation, initiates in the collectors that transport the wastewater to the corresponding treatment plants. These facilities are responsible for processing the wastewater so that it can be safely returned to the environment or potentially reused.

**Fig. 2:**
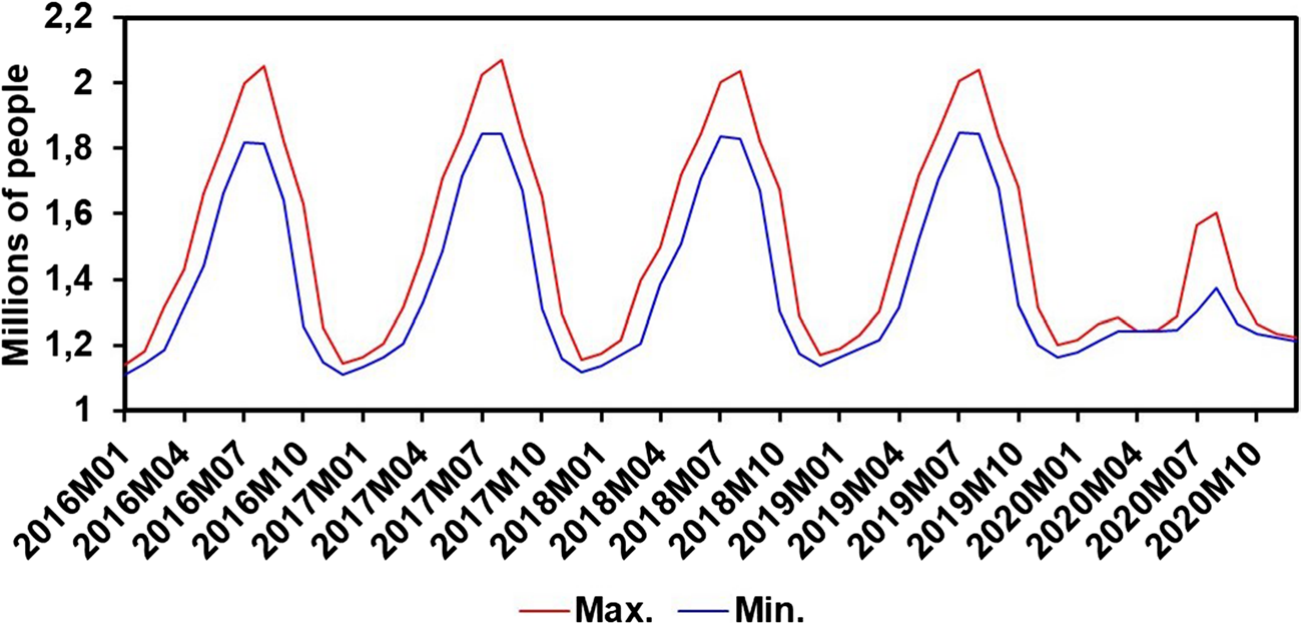
Monthly variation in the human pressure index (HPI) in the Balearic Islands from 2016 to 2020. Source: Institut d’Estadística de les Illes Balears (IBESTAT), España (CC BY 3.0)

The current state of the whole process has been compromised by the increase in population and by the seasonal nature of tourism (Martín Martín et al. 2020; Sala-Garrido et al. 2011). Tourism is a key strategy within the region, and its impact on water management is becoming more pressing (World Tourism Organization 2010). Figure 2 reveals the human pressure index (HPI), obtained by combining the resident and seasonal population figures in the study area. This index is vital for comprehending the population load and its fluctuations over time. It is worth noting that in the year 2020, which was marked by the COVID-19 pandemic, a significant deviation from the customary trend is evident, as the expected number of people was not reached. The decline is mainly attributed to the containment measures and mobility restrictions implemented in response to the health emergency, which directly influenced the demographic dynamic of the region during that period.

## Study area

The research area focuses on the Balearic Islands, an archipelago belonging to Spain in the Mediterranean Sea, which is located east of the coast of the Iberian Peninsula (Cruz-Pérez et al. 2023). It consists of two groups of islands, the Gimnesias Islands (Mallorca, Menorca and Cabrera) and the Pitiusas Islands (Ibiza and Formentera), plus several islets around these formations, in total covering an area of 4986 km^2^. In addition, its coastal bodies of water represent a surface area of 3739 km^2^, so the Balearic Islands Hydrographic Demarcation covers a total of 8725 km^2^ (Garau and Ribas 2023; Ruiz-Orejón et al. 2018).

The climate of the Balearic Islands is Mediterranean, characterised by mild winters and hot, dry summers. Summer temperatures average approximately 25–26 degrees Celsius, and in winter, they drop to 9–11 degrees Celsius on average. Rainfall tends to be more frequent in autumn and winter. The Balearic Islands also experience major episodes of torrential rain, which cause flooding, rockslides and considerable damage in the area (Grimalt-Gelabert et al. 2021; López-Bustins 2018; Reynés Vega et al. 2023).

In this context, the area is characterised by a high level of biodiversity and a fragile ecosystem, under great pressure from uncontrolled and excessive urbanisation, the concentration of tourist settlements on the coast and the seasonal nature of these tourists (Amorós et al. 2009).

**Fig. 3:**
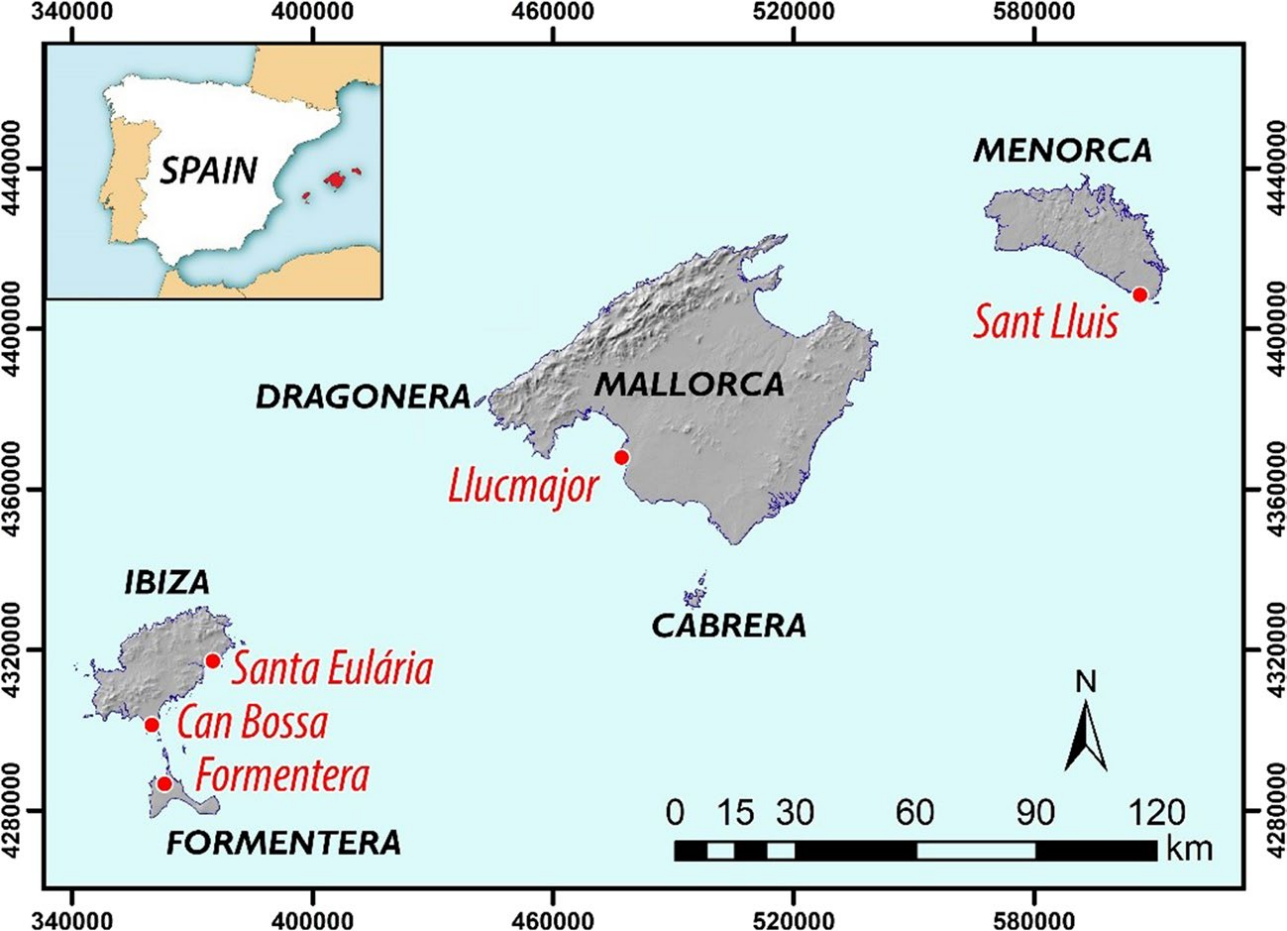
Location of the selected WWTP for the study in the Balearic Islands (Spain)

The aim of this study was to conduct an analysis of water quality during the purification process in the Balearic Islands archipelago over a period of 5 years, from 2016 until 2020. The key objective of this evaluation was to ascertain the degree to which the water quality standards set by the Balearic Island government were being appropriately maintained. To meet this aim, we chose five wastewater treatment plants that were strategically located across the archipelago, including one in Mallorca, one in Menorca, two in Ibiza and one in Formentera (refer to Fig. 3 for their exact locations).

Water quality analysis was conducted by examining several significant factors, such as flow rate, biochemical oxygen demand, chemical oxygen demand, suspended solid concentration and the presence of nitrogen and phosphorus in the water, before and after it undergoes the treatment process. This thorough methodology facilitated a comprehensive assessment of wastewater quality on all represented islands, providing valuable insights into treatment system efficacy and their impact on environmental preservation and public health in the Balearic Island region.

The limited number of WWTPs suitable for analysis was a constraint inherent in this study. Due to the uniqueness and distinctiveness of each area across the islands of the Balearic archipelago, the research team selected a representative sample of five treatment plants. This limitation highlights the necessity for perpetual surveillance and consistent monitoring in the future. This will establish a robust foundation for analysing and managing integrated wastewater treatment and sanitation infrastructure throughout the region. Additionally, gathering data over time and across various locations can greatly aid informed decision-making and the formulation of efficient public policies regarding water quality and environmental conservation in the Balearic Islands.

## Methodology

This study utilised data from the wastewater treatment plants (WWTP) of the Balearic Islands managed by ABAQUA. Data were available for both daily and monthly flow treated, as well as the conductivity measured at the inlet and outlet of the treatment plant. Additionally, measurements were taken for the 5-day biochemical oxygen demand (BOD_5_), the chemical oxygen demand (COD) and the suspended solids (SS) at both the inlet and outlet. The amounts of nitrogen and phosphorus were also measured at the inlet and outlet of the treatment plant. The aim was to assess the quality of the wastewater throughout the study period, in accordance with the regulations outlined below:

- Council Directive 91/271/EEC of 21 May 1991, concerning urban wastewater treatment
- Royal Decree Law 11/1995 of 28 December 1995 laying down the rules applicable to the treatment of urban wastewater
- Royal Decree 509/1996 of 15 March 1996 implementing Royal Decree Law 11/1995 of 28 December 1995 laying down the rules applicable to the treatment of urban wastewater
- Royal Decree 2116/1998 of 2 October 1998 amending Royal Decree 509/1996 of 15 March 1996 implementing Royal Decree Law 11/1995 of 28 December 1995 laying down the rules applicable to the treatment of urban wastewater
- Hydrological Plan of the Balearic Islands

The Balearic Water Agency manages 79 wastewater treatment plants with the following distribution (see Table 1).

**Table 1:** Treatment capacity of the WWTPs of the Balearic Water Agency in the Balearic Islands. Source: ABAQUA

Island	Number of WWTPs	Design population (e.i.)	Design volume (m^3^/year)
Mallorca	56	825,452	53,495,495
Menorca	12	254,502	15,882,975
Ibiza	10	305,530	22,293,105
Formentera	1	30,260	1,229,400
Total	79	1,415,744	92,970,975

A selection of representative WWTPs for each island was made based on several factors: (i) availability of a complete and updated series, (ii) volume of treated water, (iii) volume of reusable water and (iv) design population. According to this, we have been left with the following WWTPs (see Table 2), which are located according to Fig. 3.

**Table 2:** List of the selected wastewater treatment plants (WWTPs) in the Balearic Islands. Source: summarised from Balearic Islands Hydrological Plan 2021, ABAQUA.11-13

WWTP	Design population (e.i.)	Design flow (hm^3^/year)	Treated flow in 2015 (hm^3^/year)	Treated flow in 2020 (hm^3^/year)	Treatment	Reusable volume (hm^3^)	Place of discharge
Llucmajor-S’Arenal	79,500	5.80	2.02	1.70	Tertiary	2.02	Irrigation and outfall
Sant Lluís	15,000	1.10	0.46	0.29	Tertiary	0.46	Irrigation and hotel service
Platja d’en Bossa (Can Bossa)	41,799	2.15	1.52	0.83	Tertiary	1.52	Outfall
Santa Eulària des Riu	58,333	5.11	2.73	1.84	Secondary	2.73	Pond and outfall
Formentera	30,260	1.30	0.52	0.50	Secondary	0.52	Pond and outfall

A later stage of this study included checking whether the wastewater treatment plants (WWTPs) in question complied with the quality indicators defined in the analysis period, in accordance with the limits set by the regulations in force. Table 3 summarises the parameters that were analysed and provides an overview of their description.

**Table 3:** Summary of the parameters used for the water quality assessment

Indicator	Description of the parameter
Flow of treated water	Total volume of water that arrives at the treatment plants that is treated so that it can be returned to the environment or reused
Adequacy of the flow received to the design flow	This indicator evaluates the state of the sizing of the WWTPs, comparing the flow of municipal wastewater that arrives at each treatment plant with its design flow
Biological oxygen demand (BOD) of the treated water discharged into the sea	It measures the amount of matter that can be consumed or oxidised by the biotic community in a liquid sample. It is used to determine the degree of contamination. It is measured after 5 days (BOD_5_) and is expressed in milligrams of oxygen per litre (mg O_2_/l)
Chemical oxygen demand (COD) of the treated water discharged into the sea	This is a parameter that measures the quantity of substances that can be oxidised by chemical processes. It is used to measure the degree of contamination of organic matter, although it suffers interference with inorganic substances susceptible to oxidation. It is expressed in milligrams of oxygen per litre (mg O_2_/l). Its value is always higher than the biological oxygen demand (BOD)
Suspended solids in the treated water discharged into the sea	This represents the set of small solid particles that are dissolved in a liquid. It is an analytical parameter used to determine the quality of treated water and is expressed in milligrams per litre (mg/l)
Total nitrogen of treated water discharged into the sea	Total nitrogen is the sum of inorganic nitrogen forms—nitrate (NO3-), nitrite (NO3-) and ammonium (NH4+)—and organic nitrogen
Total phosphorus in treated water discharged to the sea	Phosphorus is another essential nutrient for life

In addition to the evaluation of the quality indicators, the disposal performance of each parameter was calculated, allowing a more detailed and comprehensive monitoring of compliance.

With regard to the flow adequacy indicator, whether the flow received by the treatment plants studied was lower than the design flow established for each WWTP in each month between 2016 and 2020 was checked. All cases of non-compliance with the design flow were recorded in detail. For a WWTP to be considered undersized, a criterion of exceeding the design flow for more than two consecutive months was established.

With regard to the other indicators of treated water quality, a separate analysis was carried out to verify compliance with the defined requirements, in terms of exceeding the maximum permitted concentration and achieving the minimum percentage of reduction, in accordance with the national regulation.

It is important to note that all instances of non-compliance were recorded for the parameters biochemical oxygen demand (BOD), chemical oxygen demand (COD), suspended solids (SS) (see Table 4), total nitrogen and total phosphorus (see Table 5). These were then compared with the maximum concentration established by RD 509/1996 and the percentage of reduction. In order for a wastewater treatment plant to be considered non-compliant for any of these parameters, a criterion was established requiring more than three measurements exceeding the established limits in more than 16 measurements and two measurements in less than 16 measurements. This rigorous methodology provided a sound basis for assessing and monitoring the performance of STPs against quality and efficiency standards.

**Table 4:** Parameters, maximum permitted concentrations and minimum percentages of reduction of treated wastewater established in state regulations (RD 509/1996). *Compliance with the suspended solid parameter is voluntary according to these regulations

Parameter	Maximum permissible concentration	Minimum percentage of reduction
Biological oxygen demand at 5 days (BOD_5_)	25 mg O_2_/l	70–90%
Chemical oxygen demand (COD)	125 mg O_2_/l	75%
Suspended solids (SS)*	35 mg/l	90%

**Table 5:** Parameters, maximum permissible concentrations and minimum reduction rates of treated wastewater discharged into eutrophication-sensitive areas according to the number of equivalent inhabitants (e.i.) of the treatment plant (if more than 10,000 e.i. are exceeded). The N and P concentration values constitute annual averages of the samples obtained during this period

Parameter	Concentration	Percentage of reduction
P (10,000–100,000 e.i.)	2 mg/l	80%
P (> 100,000 e.i.)	1 mg/l	85%
N (10,000-100,000 e.i.)	15 mg/l	70–80%
N (> 100,000 e.i.)	10 m/l	70–80%

Failure to comply with the parameters set out in Table 4 would be illegal, as they are mandatory, but the limits for suspended solids are recommended values and are therefore voluntary. The values included in Table 5 refer to the nutrient limits in sensitive areas of eutrophication, since the treatment plants analysed exceed 10,000 equivalent inhabitants (e.i.).

## Results

### Treated water flow rate

**Fig. 4:**
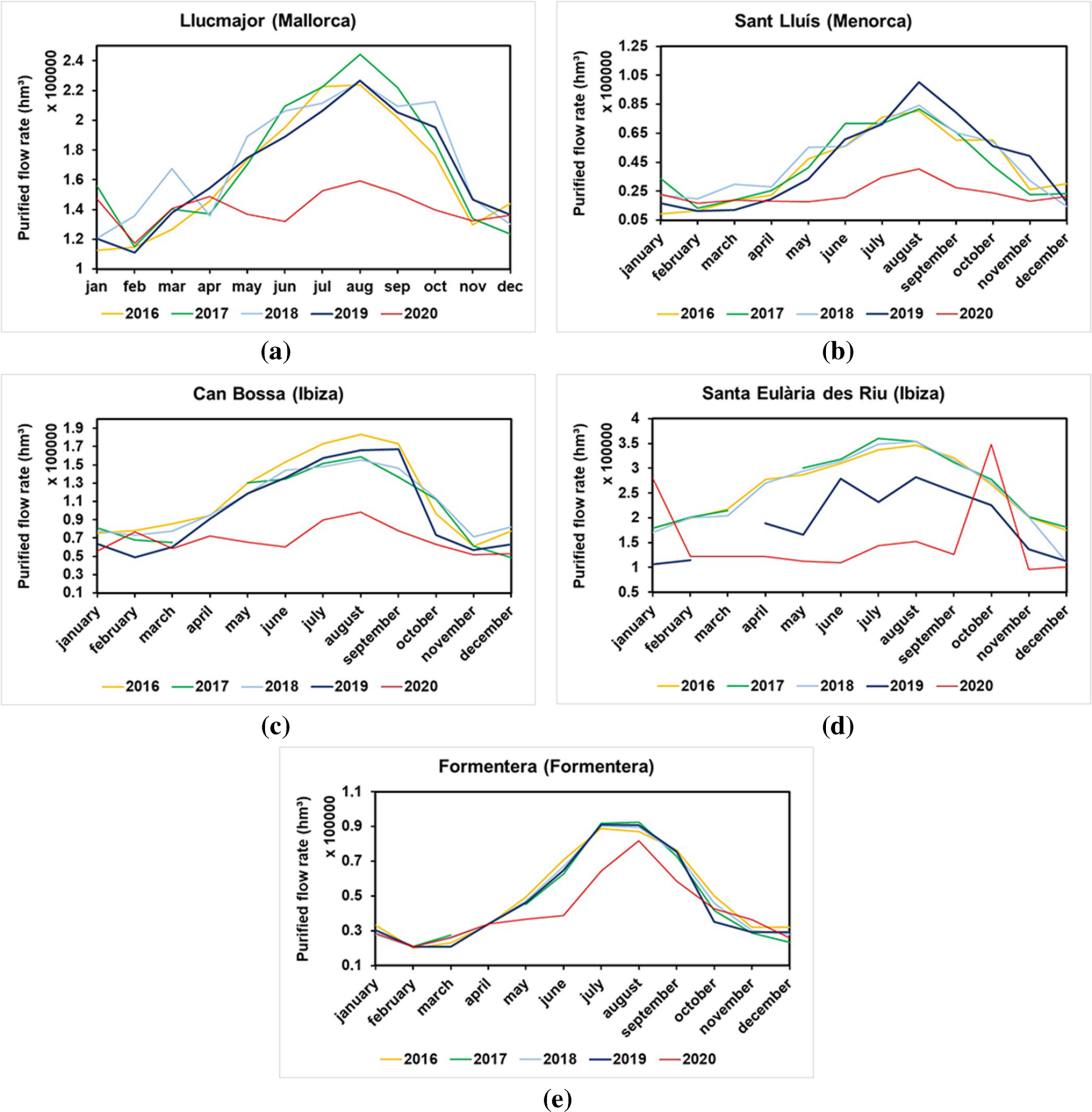
Monthly flow treated at the Llucmajor (**a**), Sant Lluís (**b**), Can Bossa (**c**), Santa Eulària des Riu (**d**) and Formentera (**e**) WWTP during the study period

The results obtained clearly show the influence of the seasonal nature of tourism on the volume of water treated in the treatment plants. During the summer months, the volume of water treated rises considerably, sometimes even doubling, such as in the case of Llucmajor, or tripling, as in the case of Formentera. The volume of treated water in 2020 varies considerably, largely due to COVID-19 pandemic, as illustrated in Fig. 4.

In general, all the WWTPs analysed met the requirement of not exceeding the design flow for more than two consecutive months. However, there were two exceptions. An exceedance was registered at the Can Bossa plant in August 2016, while a comparable non-compliance incident occurred at the Sant Lluís plant in August 2019. This coincided with the peak tourist period at both sites.

### Indicator of the adequacy of the flow received to the design flow

**Fig. 5:**
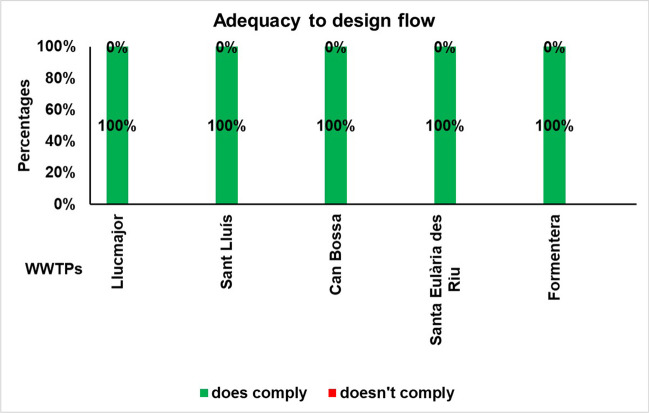
Percentage of annual compliance of adequacy to design flow at the study WWTPs for the period 2016–2020

For the analysis of this parameter, a compliance criterion was set that limits negative measurements to a maximum of two per year. The reviewed wastewater treatment plants (WWTPs) do not exhibit any annual non-compliance with the design flow according to this criterion. Thus, it can be inferred that they cannot be regarded as undersized. During the investigation, there were two instances where certain WWTPs exceeded their design flow rates. In August 2016, the Can Bossa WWTP recorded excess flow, while in August 2019, a similar situation occurred at the Sant Lluís WWTP. It should be noted that while non-compliance occurred during a particular month, an annual analysis (refer to Fig. 5) indicates overall compliance with this indicator, as the established threshold was not exceeded on more than two occasions out of a total of 12 measurements.

It is worth noting that, in the majority of the municipalities in the Balearic Islands, rainwater is not separated from wastewater. This could result in potential discharge of untreated water due to the surge in peak flows brought about by heavy rainfall events, which are frequent in the region. Moreover, it is crucial to take into account that the frequency of these weather incidents is anticipated to escalate, as per the IPCC data. Therefore, this highlights the significance of ensuring consistent monitoring of tis parameter and implementing appropriate preventative measures in the management of water resources in the region.

### Biological oxygen demand (BOD) of treated water discharged to the sea

According to current regulations, the biological oxygen demand (BOD) at the outlet of the treatment plant must not exceed 25 mg O_2_/l or a minimum reduction of 70 to 90% of the BOD at the inlet of the WWTP must be achieved. Fulfilling one of these two standards is obligatory to comply with the regulations.

Over the study period, three inconsistencies were detected, specifically in 2016 at the Platja d’en Bossa WWTP and in 2017 and in 2018 at the Santa Eulària des Riu WWTP, with regard to the concentration limits. No instances of non-compliance were found in terms of reduction percentages, and the WWTPs studied consistently met the minimum reduction values.

**Fig. 6:**
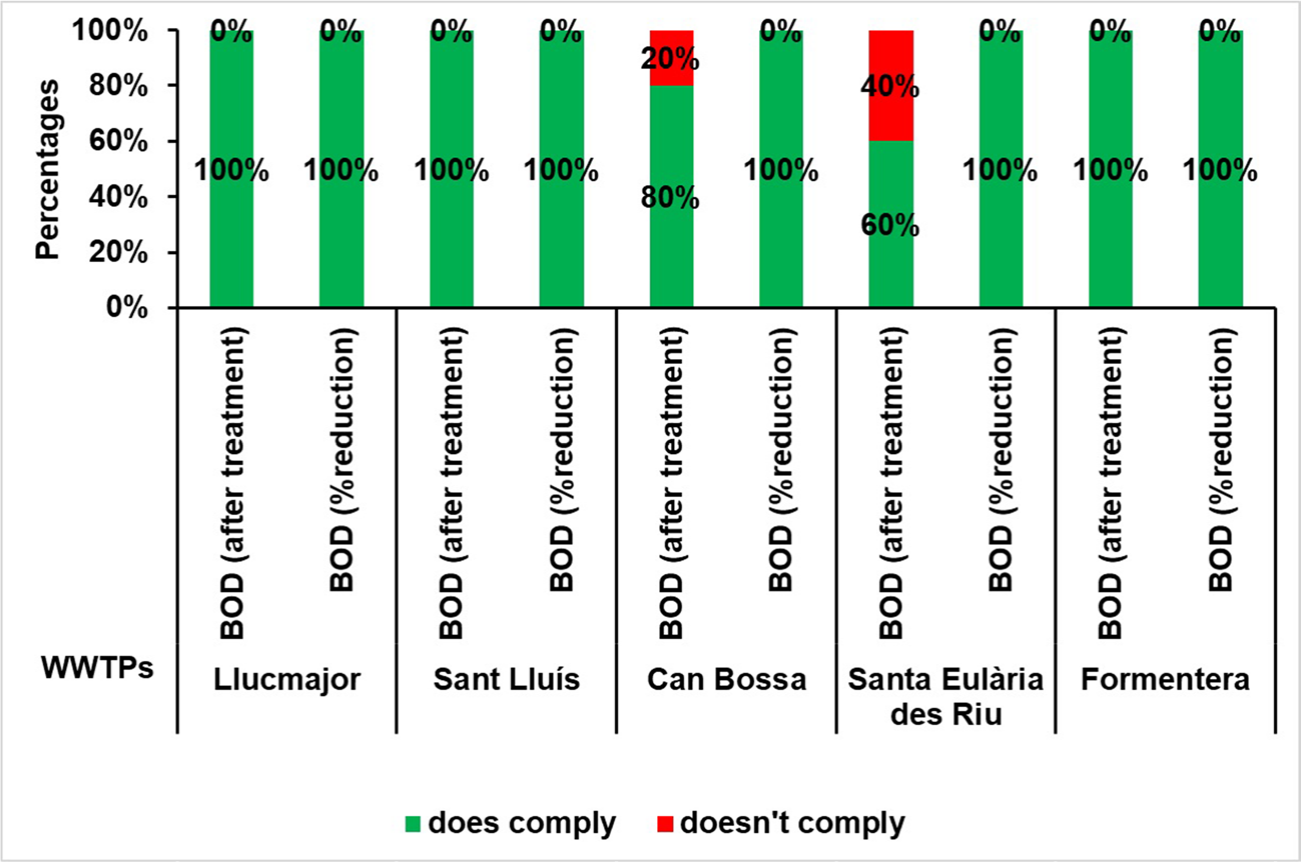
Percentage of annual compliance of BOD according to RD 509/1996 at the study WWTPs for the period 2016–2020

Therefore, in the period from 2016 to 2020, 1% of the measures exceeded the threshold concentration limit and 0% if we consider the percentage of reduction, but they comply with the law, as they meet at least one of the requirements (see Fig. 6). With these values, we can infer that the organic matter load of the treated water in these treatment plants is significantly reduced with respect to the input value.

### Chemical oxygen demand (COD) of treated water discharged to the sea

This parameter provides an insight into the organic load of the treated water, similar to the previous indicator. The regulations dictate that the COD must not exceed 125 mg O_2_/l or a minimum reduction of 75% must be attained in comparison to the plant’s input value. These requirements must be met to comply with the standard. Practically identical to BOD, there were two incidents of non-compliance regarding the concentration limit at the Santa Eulària des Riu WWTP in both 2017 and 2018. Additionally, one instance of non-compliance was reported in 2016 at the Platja d’en Bossa WWTP in terms of percentage reduction. This proportion of non-compliance regarding the concentration and percentage reduction of BOD and COD indicates that the treatment plants can significantly reduce these two indicators, but that the incoming flow has an excessive load of organic matter.

**Fig. 7:**
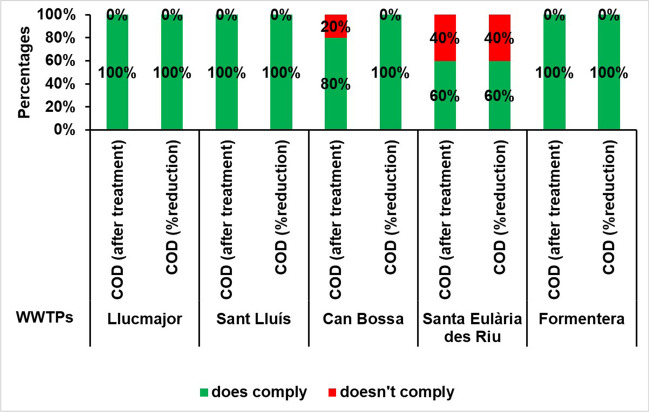
Percentage of annual compliance of COD according to RD 509/1996 at the study WWTPs for the period 2016–2020

Consequently, 0.7% of the measurements during the study period fail to comply with the maximum concentration threshold, while 0.3% do not satisfy the minimum 75% COD reduction requirement. However, this is not unlawful, as at least one of the criteria is fulfilled in all instances (refer to Fig. 7).

### Suspended solids in treated water discharged into the sea

Compliance with this indicator is not legally required, although non-compliance with the values set out in Royal Decree 509/1996 is discouraged. The regulation advises against exceeding suspended solid concentration beyond 35 mg/l or achieving a minimum reduction rate of 70 to 90%.

**Fig. 8:**
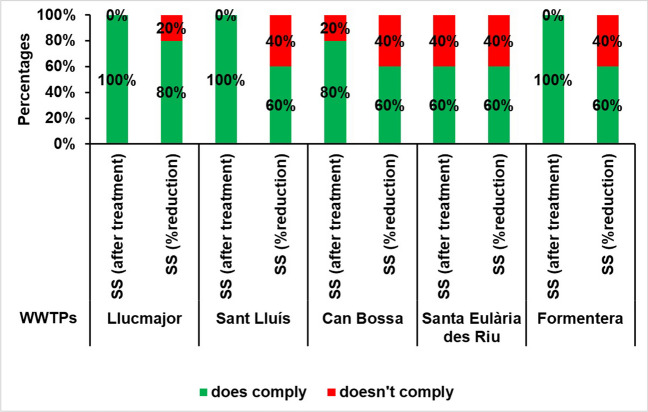
Percentage of annual compliance of suspended solids according to RD 509/1996 at the study WWTPs for the period 2016–2020

It is important to note that the percentage reduction is more frequently exceeded than the concentration threshold. A total of nine annual breaches were observed for the percentage of reduction across all the WWTPs studied. Specifically, these breaches occurred in Llucmajor in 2018, in Sant Lluís in 2017 and 2019, in Santa Eulària des Riu in 2017 and 2018, in Platja d’en Bossa in 2016 and 2019 and in the WWTP of Formentera in 2019 and 2020. The concentration threshold was breached three times during this period at the Santa Eulària WWTP reporting it in 2017 and 2018 and Can Bossa in 2016 (see Fig. 8).

### Total nitrogen in treated water discharged into the sea

This parameter is important because the increased nutrients discharged into the sea cause an acceleration in the production of algae, an increase in the accumulation of organic matter and an excessive consumption of oxygen. As a result, adverse impacts befall the marine environment, causing displacement of specific species from the region and bringing about a complete metamorphosis in the ecosystem.

**Fig. 9:**
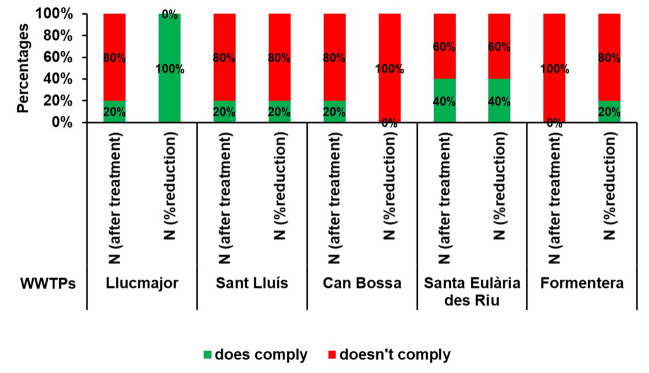
Percentage of annual compliance of total nitrogen according to RD 509/1996 at the study WWTPs for the period 2016–2020

RD 509/1996 establishes the limit of nitrogen concentration at 15 mg/l at the outlet of the treatment plant and recommends that a minimum of 70% reduction be achieved with reference to the inlet measurement. In numerous instances during the study period, the wastewater treatment plants failed the recommendations of the RD regarding this parameter, as demonstrated in Fig. 9.

In 2020, the final year of study, the recommended values were not reached solely at the Can Bossa WWTP in reduction percentage and at the Formentera WWTP in nitrate values after treatment. Despite this, the maintenance and modernisation activities implemented at the treatment plants during that period were successful, as data analyses revealed improvements at all the evaluated stations.

### Total phosphorus in treated water discharged into the sea

With regard to phosphorus, the RD recommends that areas prone to eutrophication should limit their concentration to less than 2 mg/l for WWTPs with a capacity of between 10,000 to 100,000 population equivalents and less than 1 mg/l for WWTPs with a capacity exceeding 100,000 population equivalents. Additionally, the RD mandates a minimum reduction of 80% of the phosphorus load in the water that these WWTPs receive.

**Fig. 10:**
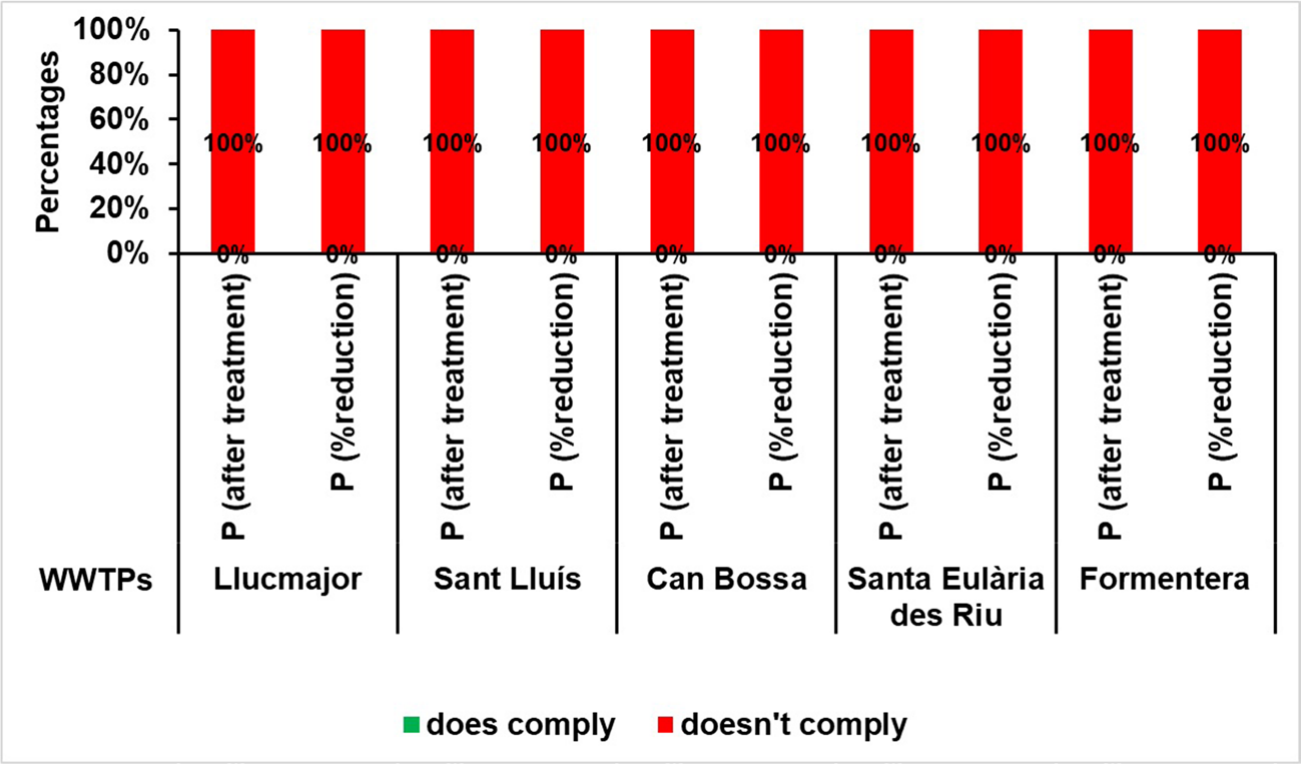
Percentage of annual compliance of total phosphorus according to RD 509/1996 at the study WWTPs for the period 2016–2020

Throughout the 5-year study period, the WWTPs under investigation repeatedly breached the established limit, failing to comply on every single occasion (refer to Fig. 10). This is a cause for concern, since the high levels of phosphorus in environmentally sensitive areas prone to eutrophication, together with the presence of nitrates, may lead to a surge in nitrogen-fixing microalgae that could trigger episodes of phytoplankton blooms that are linked to oxygen deprivation.

These values indicate that further improvement and modernisation is necessary to optimise the treatment process in these WWTPs.

## Discussion

Tourism has a substantial effect on the Mediterranean Sea (Borja et al. 2016; Cole 2012; Coll et al. 2010; Pınarbaşı et al. 2017), which is globally recognised as one of the most significant tourist destinations. The constant influx to this region is predominantly driven by the richness and variety of its natural landscape and heritage (Cruz-Pérez et al. 2023; Eurostat/European Commission 2009; Valdivielso and Moranta 2019). Tourism in the region demonstrates a pronounced seasonality, as outlined in the European Environmental Assessment (EEA 2003), due to the significant concentration of visitors at specific times and locations. This generates direct impacts on both the local population and the natural environment.

Tourism has been recognised as one of the primary factors influencing the Mediterranean coasts, particularly regarding wastewater generation. Recent years have seen a steady rise in coastal tourism, and this has had a noticeable impact on marine water. Consequently, significant investments have been made in technology and financial resources to enhance wastewater management, thereby improving the environment’s quality (Mallorquí et al. 2015). One of the main environmental hazards associated with tourism industry is the unregulated release of untreated wastewater into marine ecosystems and the sea, leading to the contamination of vital water resources (Stonich 1998).

In addition, wastewater production can result in local imbalances within the marine ecosystem, particularly (i) the existence of insufficiently dimensioned treatment plants or those that are technologically underdeveloped, (ii) seasonal irregular dumping and factors related to global warming, like the increasing sea level (which causes flooding and damage to sewage facilities, as noted by Phillips et al. (2015)), (iii) the diversity of stakeholders and practices in the tourism domain, (iv) the scarcity of high technology monitoring tools (e.g. distance control, computerised nutrient analysis) and cost-effective building techniques, (v) the lack of compliance monitoring and policy enforcement and (vi) insufficient basis for analytical decision-making. Water consumption as well as wastewater production may differ according to prevailing land uses and associated densities (i.e. camping sites, hotels, resorts, flats, cottages, residences), two key variables in assessing the impacts of tourism on the economy, society and the environment (Gössling 2002; Hall and Gössling 2006). Furthermore, tourism destinations exhibit varying business profiles as determined by factors such as seasonality, the proportion of holiday homes and day visitors to its resident population, floating population and industrial and commercial demand (Torres-Bagur et al. 2020).

Increased tourism has led to a rise in the generation and discharge of wastewater into the sea, with potential repercussions such as elevated nutrient levels and resulting eutrophication. This may have detrimental effects on both local biodiversity and water quality (Beck et al. 2011; Campagne et al. 2015; Milanese et al. 2011; World Tourism Organization 2010).

It should be noted that the tourism industry has a twofold effect (Chao and Chao 2017; Gössling and Peeters 2015; Phong and Van Tien 2021): it can offer chances to advance and set back commercial ties, income from foreign exchange transactions, business diversification, growth in earnings, more employment and the fight against poverty; and it can also jeopardise cultural and environmental resource conservation, key elements to attracting tourists. Consequently, there is widespread agreement that national policies and measures are necessary to minimise the negative impacts of tourism on both the natural environment and cultural heritage (Llamas et al. 2015).

The study results reveal a specific concern regarding the elevated levels of suspended solids, nitrates and phosphorus in the water. In general, the presence of nitrates in water is associated with agricultural land usage, a recurring trend in various islands and regions due to insufficient nutrient and fertiliser management (Bruthans et al. 2019). This, in turn, requires tertiary treatments to eradicate these chemicals, leading to increased costs (Costa et al. 2021; Zhang et al. 2019). A recent study in *El Hierro* (Canary Islands) (Gasco et al. 2023) demonstrated good practices in nutrient management, where only the necessary amount of nutrients is provided to the soil for the absorption by plants. As a result, pollution is avoided, and the need for expansion of tertiary treatments in the WWTPs is eliminated.

Some authors have highlighted the tourism sectors and urged the authorities to mitigate the environmental consequences of their actions (Gabarda-Mallorquí et al. 2018). The expenditures associated with water management have generally been sustained by taxpayers as well as the private sector, which views it as a necessity for maintaining current services and initiating new ones. To date, the prioritisation of measuring and assessing the impact of tourism aligns with the Mediterranean Strategy for Sustainable Development (MSDS) and the global objectives of the 10YFP Sustainable Tourism Programme (SDG 12.1), which advocate for a comprehensive approach to environmental, social and economic factors (Antipolis 2009). This initiative aims to enhance and broaden established practices, fortify collaborations and cultivate fresh strategies in order to speed up the shift towards more eco-friendly manufacturing and consumption.

Integrated water resources management (IWRM) is a useful tool for promoting efficient water usage (Santamarta et al. 2022) while concurrently supporting the UN Sustainable Development Goals. The European Union has reinforced its commitment with the European Sustainable Development Strategy to provide policies and activities to (i) ensure the protection and improvement of the environment; (ii) promote a democratic, inclusive and cohesive society; (iii) foster economic prosperity; and (iv) support sustainable development worldwide (Commission of the European Communities 2005). Water management and protection covers all key objectives in a cross-cutting manner (climate change, energy, transport, consumption and production, natural resources, public health and global poverty and development) (Gooch and Stålnacke 2010).

## Conclusions

The study examined water quality in treatment plants across diverse locations in the Balearic Islands, assessing parameters established by national regulations. Improvements were noted in all parameters during the period analysed, indicating the efficacy of measures implemented in preceding hydrological plans. The seasonal nature of tourism influences the generation of wastewater, with a substantial surge during the high season. In certain plants, the design limit was exceeded solely during the high season. Occasional deviations occurred in certain parameters, including BOD, COD, SS, N and F. Albeit pollutants are removed efficiently, nitrogen and total phosphorus values in the effluent fall short of the quality standards. To sum up, regulatory compliance is met, but seasonal tourism has a substantial impact on treated water. Further research is recommended, which includes conducting a comparative analysis with data obtained from 2021 and 2022. Additionally, exploring sources and optimising options for reducing nitrogen and phosphorus pollutant loads is crucial.
